# An Appeal for Higher Art in Amalgam Fillings

**Published:** 1914-03-15

**Authors:** Wm. R. Pond

**Affiliations:** Rutland, Vt.


### AN APPEAL FOR HIGHER ART IN AMALGAM FILLINGS.

BY WM. R. POND, D. D. S., RUTLAND, VT.

The following is an extract from an article published in the January *Items of Interest.*

It is said that about seventy-five per cent. of our filling work is done with amalgam. Why, then, devote all our attention to perfecting a technique for the remaining twenty-five per cent? Let us have a definite technique for amalgam work, and let us keep on perfecting this until we reach as near a uniform result of excellency as it is possible to attain.

With this idea of evolving a better technique for amalgam work the writer will make some suggestions and give some description of the work as he sees it. It is to be hoped these suggestions may give rise to enough discussion to bring improvement where it is so much needed.

Regarding the alloy to be used much might be said, but as this subject is being widely discussed and investigated, we shall undoubtedly have more uniform formulas in the near future. Dr. Marcus L. Ward, in a recent paper, makes the statement that he is convinced the reputable supply houses are making honest effort to put good alloys on the market. This is certainly a reassuring report from a man of standing. The writer can only say, that having used one alloy for fourteen years, he knows it to be a uniform product, and to his familiarity with the working properties of this alloy he attributes no small share of his success with amalgam as a filling material. This is a so-called quick setting alloy, but is what the manufacturers term "slower setting."

Cavity preparation for amalgam work should be similar to that for inlays. The margins should be clean cut, polished and well extended, and in their preparation the mounted carborundum stones will be found invaluable. In deep cavities, build up with cement to obtain a typical preparation with flat seat. Little or no undercut is necessary if the first layer of amalgam is cemented in as is the writer's invariable custom. The zinc cements are used, except in a few cases, where the oxy-phosphate of copper seems advisable.

The matrix is always indicated where any contour is to be made. The Ivory matrix holders, with thin steel matrix band, or those cut from celluloid strips are very satisfactory when used in conjunction with wedges placed at the cervix. In extreme restorations, make a soldered matrix of thin orthodontia band material, made either by "pinching" and soldering, as the orthodontist makes a plain band, or cut and soldered from a wire measurement of the tooth. These matrices can be accurately fitted, contoured and wedged, and at the desired time can be quickly split and removed.

If a large restoration is to be made, study carefully the occlusion and the movements of the madible, and if necessary, take a wax or modeling compound "bite" and run plaster casts for study models. A hasty filling of temporary stopping, allowing the patient to close the teeth together while the material is still plastic, will show any abnormal conditions of "bite," which can be allowed for in the amalgam restoration. In amalgam work the rubber dam is seldom indicates, as so many of the fillings extend below the gum. Cotton rolls and the saliva ejector are perfectly edequate except in rare cases.

Dry out the cavity with warm alcohol, and then, mixing a white cement to about the same consistency as for setting inlays (do not use a hydraulic cement, as all moisture is excluded), and with a fine instrument line the cavity with the cement. Next apply the matrix, if a compound cavity is to be filled. In difficult cases the matrix should be applied before the cement lining. Now mix the amalgam, everything being in readiness for rapid work, or have the assistant do the mixing while the matrix is being applied. Pack the amalgam, using large pieces and ample pressure, working the first pieces into the cement, covering all surfaces, floor, walls and margins, and wiping away the excess of cement. Then fill slightly to excess, using large pieces and sufficient pressure to produce a homogeneous mass. Contour ample cusps. Except in extreme restorations, where it has been decided to leave a soldered matrix, to be removed at a future sitting, the matrix is immediately removed, being withdrawn from the side, either buccally or lingually, as seems advisable. The margins which were hidden by the matrix are now carefully examined and if any lack of contour shows, a small quantity of filling material may be added. The occlusal surface of the filling is now roughly "troughed out" and wiped over with a pledget of cotton. This gives a frosted surface which will show every contact point when the teeth are brought together. The patient should be told to "close" carefully (and this should be done as soon as possible, and before the filling material has commenced to crystalize), using very little pressure. The occlusal surface is gradually worked down, noting the contact each time of closing. As soon as the teeth can be brought comfortably together, the movements of the mandible are noted and allowed for when necessary.

The filling is now gone over with a large ball burnisher and allowed to set for about ten minutes before further work on it is done. At this time the cervical margins are carefully examined, and as the filling material is now at a more granular stage, any small particles which may have been forced under the gum may be removed with a fine-pointed scaler, and these margins rubbed over carefully with a file (made dull), such as is used in the sets of scaling instruments.

We now turn to the carving of the occlusal surface. The amalgam is now at a stage where it gives a crinkly sound on being burnished, and burnishing gives it a polished surface like nickel. Burnish toward the margins, using a large ball instrument first. Using successively smaller ball burnishers, work out roughly the lines, until with an Ash No. 1 plastic instrument (the fine ball end) the grooves are fairly well outlined. Next reverse the instrument and deepen the lines with the small thin blade. Now with a fine pointed instrument (a pair of very fine-pointed scalers, Grafarth No. 6 and 7 are about right), the grooves and sulci can be finished as effectively as can be done in a wax pattern for a gold inlay.

Absolutely the last finishing work to be done on the filling is to obtain proper contact points approximately. By this time the amalgam is quite hard and brittle and a fine strip will remove none of the material. Pass a Gordon White separating saw (it will go through a narrower space than even a floss silk) between the fillings, or between filling and tooth, as the case may be, and then with a very thin linen strip with no grit on it, held taut by winding one end around the beaks of pliers and the other held in the hand, polish between the teeth, making a smooth contact point, but removing no filling material. Bring the strip down to the cervical margin, polish beneath the gum, and the filling is done unless it is desirable to polish some of the margins at a later sitting. This is seldom necessary or desirable, as the finish obtained by burnishing is most attractive. In extreme restorations, where the matrix is left on until another sitting, the later polishing with discs and strips is a necessity. The occlusal carving in these fillings is done as described before.

Where approximal fillings are inserted, one filling, either mesial or distal, as convenient, must be finished at its approximal contact point (and in this case the wait need not be so long before finishing here) when the matrix is inserted for the remaining filling and this is contoured to meet the contact point planned for the first. Positively two mixes of amalgam should be made and the first filling will be in condition to be carved by the time the second has been inserted. When a number of amalgam fillings are to be made for a patient, practically no time need be lost in waits for carving and finishing as the work may be so arranged that several fillings can be carried on together.

The so-called quick setting alloys take many hours to reach their maximum strength, but two or three hours puts the fillings made with these alloys beyond danger of breakage of cusps or contour, provided proper occlusion has been secured and sufficient allowance made for the movements of the mandible. The greatest danger of breakage is within the first half hour.

A few warnings regarding amalgam work may not be amiss. Do not try to make contours without a matrix and do not try to make too many fillings with one "mix." Do not polish with a disc until several hours after the filling has been inserted, and do not use a cutting strip on the approximal contact points. Do not make the mix so dry that it is crumbly, as it cannot be contoured, nor made a homegeneous filling under these circumstances. Above all, do not fail to give the same serious attention to detail in amalgam work that you would to a gold inlay, and do not fall short on the foundation work—a proper cavity preparation.

In trying to reproduce natural tooth forms and correct occlusal surfaces, one must know dental anatomy, and have an idea of what constitutes normal occlusion. He must know just where belong the cusps and planes, the grooves and sulci. Surely it is necessary to have an ideal in mind before we try to create mechanically, and I may add, no man of judgment would put fifteen-year-old cusps on a sixty-year-old tooth, nor should he prepare youth for old age by building a flat-worn cusp on the tooth of a child of fifteen.

In closing let me say, that amalgam filling work not only *will* survive, but it *should* survive. It is the "hope" of the fifty million carious teeth in children's mouths to-day, provided we, as dental operators, restore these teeth to the form and efficiency which Nature intended them to have. Are we equal to the task of fifty million gold inlays, and are the children equal to it? I think not. The task looms big enough spelled in amalgam restorations.

